# Sulfisoxazole does not inhibit the secretion of small extracellular vesicles

**DOI:** 10.1038/s41467-021-21074-x

**Published:** 2021-02-12

**Authors:** Pamali Fonseka, Sai V. Chitti, Rahul Sanwlani, Suresh Mathivanan

**Affiliations:** Department of Biochemistry and Genetics, La Trobe Institute for Molecular Science, La Trobe University, Melbourne, VIC Australia

**Keywords:** Cancer, Breast cancer

**Arising from** Eun-Ju Im et al. *Nature Communications* 10.1038/s41467-019-09387-4 (2019)

Recently, the study by Im et al. focused on blocking the release of extracellular vesicles (EVs) from cancer cells, as a strategy to block metastasis, by deploying a drug repurposing screen. Upon screening the library of FDA approved drugs in breast cancer cells in vitro, the authors reported the ability of the antibiotic Sulfisoxazole (SFX) in inhibiting EV biogenesis and secretion. SFX was also effective in reducing breast primary tumor burden and blocking metastasis in immunocompromised and immunocompetent mouse models. As we seek a compound to block EV biogenesis and secretion in our current in vivo studies, we intended to use SFX and hence performed in vitro characterization as the first step. However, treatment of two cancer cells with SFX did not reduce the amount of EVs as reported by the authors.

Over the last two decades, extracellular vesicles (EVs) have been implicated in intercellular communication and utilised as drug delivery vehicles and as reservoirs of disease biomarkers^1–5^. As they continue to garner interest, several seminal studies have established that EVs regulate various pathophysiological processes in favour of cancer progression, including remodelling the tumour microenvironment, immune evasion, coagulation, vascular leakiness, establishing the pre-metastatic niche, tropism for metastasis and transfer of chemoresistance^6–11^. Hence, there is growing interest in blocking the release of EVs and limiting their systemic circulation as a novel therapeutic avenue to treat cancer^12^. As anti-metastatic therapies are scarce, it is speculated that FDA approved drugs that target EVs could possibly fill the void.

Recently, the study by Im et al. focused on blocking the release of EVs by cancer cells, as a strategy to block metastasis, by deploying a drug repurposing screen^13^. The rationale was to screen the existing FDA approved library to identify drugs which can inhibit EV biogenesis or secretion with the obvious advantage of known mode of action, efficacy and toxicity profile and hence has the potential of immediate clinical utility. Upon screening the library of FDA approved drugs in metastatic breast cancer cells in vitro, the authors reported the ability of the antibiotic sulfisoxazole (SFX) in inhibiting EV biogenesis and secretion. The authors also reported that SFX was effective in reducing breast primary tumour burden and blocking metastasis in immunocompromised and immunocompetent mouse models. SFX was proposed to target endothelin receptor A (ETA) previously in 1994^14^ and hence the authors validated that SFX targets ETA which can positively regulate EV biogenesis and secretion. The findings in this study thus present SFX as a potential novel EV-targeted therapeutic alternative. As a group interested in EVs, the outcomes proposed in the study were encouraging and attractive as FDA approved drugs targeting EV release are limited. Recently, Datta et al. also performed a repurposing screen to identify drugs that modulate the release of EVs in prostate cancer cells^12^. However, the identified drugs are yet to be tested in vivo.

As we seek a compound to block EV biogenesis and secretion in our current in vivo studies, we intended to use SFX and hence performed in vitro characterization as the first step. However, treatment of 4T1 breast cancer cells with SFX did not reduce the amount of EVs as reported by the authors. We acknowledge the fact that our EV isolation protocol^15^ was different from the study^13^ (Fig. 1 Mathivanan laboratory protocol) and hence could have attributed to the varied results. In order to rule out the possibility of variations in the method of EV isolation or cell-type dependency, three researchers exactly followed the protocol employed by the authors (Fig. 1 protocol 1 and 2) to isolate EVs from two different cell types (4T1 and MDA-MB231) that were used by the authors (see ‘Methods’). Protocol 1 (Fig. 1b) is exactly the same protocol reported in Im et al.^13^ while protocol 2 was provided by the authors when contacted by us while attempting to repeat their experiments. For protocol 2 (Fig. 1), EVs were isolated with slight modifications from protocol 1 (differential centrifugation 2500 × *g* for 15 min and sEV incubated for 1 h at 4 °C). The modifications in protocol 2 as compared to protocol 1 is denoted with * in Fig. 1b.

**Fig. 1:**
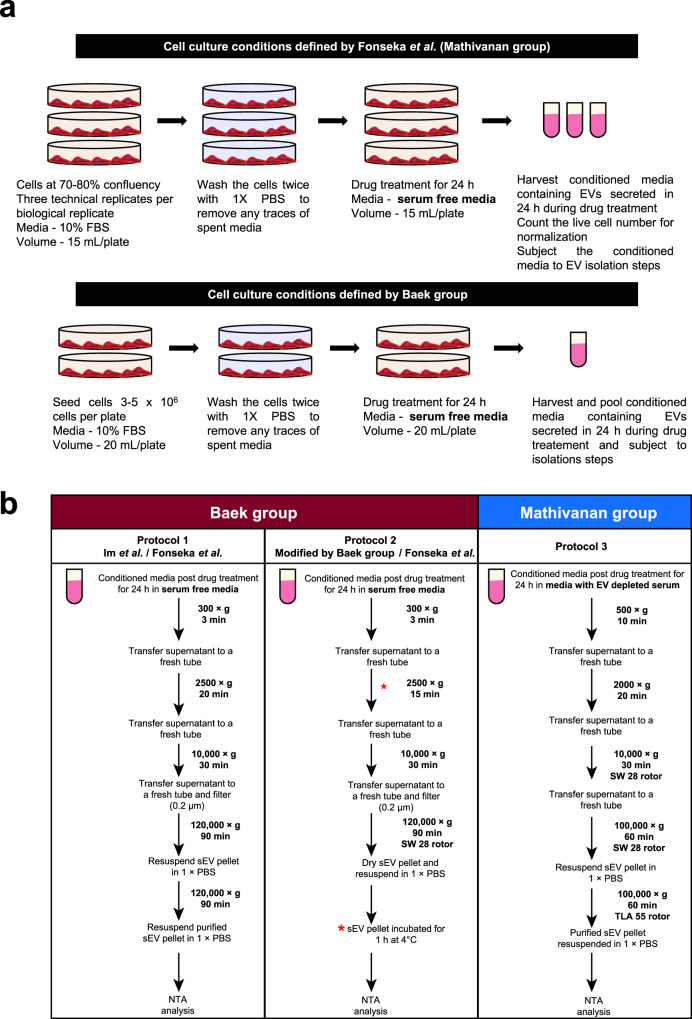
Schematic representation of cell culture, drug treatment and sEV isolation by two groups. **a** Schematic representation of cell culture and drug treatment approaches. The cells were seeded and allowed to grow to 70–80% confluency in media supplemented with FCS. Upon attaining desired confluency, cells were washed with 1× PBS and subjected to drug treatment for 24 h in serum-free media. At treatment endpoint, conditioned media was harvested and subjected to sEV isolation. Live cell number was determined to normalise particles released to cell number. Similarly, Baek group seeded cells in a pre-defined range and treated them with drug in serum-free media. **b** Protocol 1, schematic flow diagram depicting the methodology of sEV isolation and analyses as defined by Baek group in the initial publication (Im et al.^13^) and followed by Fonseka et al. (this study). Protocol 2, schematic flow diagram depicts the methodology of sEV isolation and analyses as reported by Baek group when approached. The variations in the methodology from the original manuscript (Im et al.) is marked (*). Protocol 2 was also adapted by Fonseka et al. to include the variations (*) suggested by Baek group. Protocol 3, standardized and optimized method of sEV isolation in EV depleted serum used in Mathivanan laboratory was also used to collect sEVs post treatment with SFX.

All these assays were performed with three technical replicates (same batch of cells seeded in three different plates) for every biological replicate. Consistent with our previous observations, treatment of the cancer cells with varying concentrations of SFX (50, 100 and 200 µM) did not impede the release of EVs while the positive control ceramide inhibitor GW4869, at low concentration (5 µM), inhibited EV secretion. Upon EV isolation, we quantified the protein amount, particle number and performed western blotting for EV enriched proteins (TSG101, Alix), all normalised to equal cell number (Fig. 2 and Supplementary Fig. 1). Contrary to the authors claim of SFX treatment led to a 3-fold decline in EV particle number, we observed a significant increase in particle number upon SFX (200 µM) treatment.

**Fig. 2:**
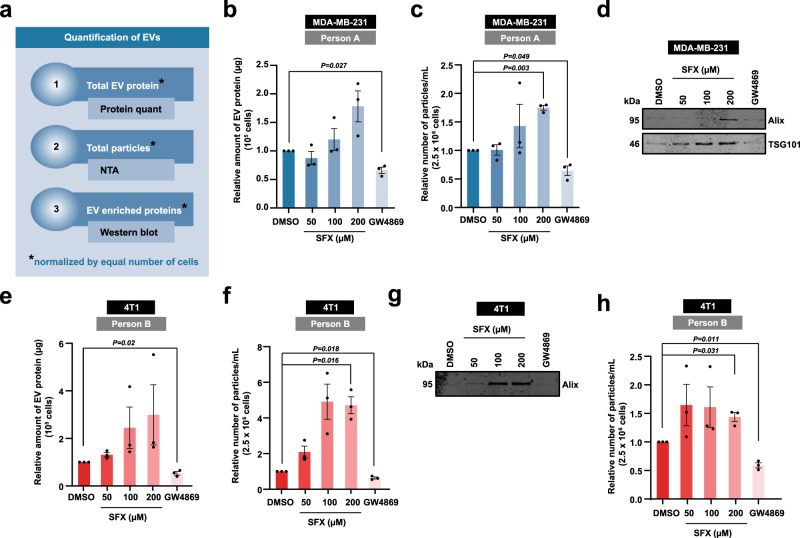
Quantification of EVs released by cells with or without SFX and GW4869. **a** Schematic of EV quantification by three different methods. Firstly, the total EV protein amount was quantified and normalised to equal number of live cells. Secondly, nanoparticle tracking analysis (NTA) was performed to quantify the total number of particles normalised to equal number of live cells. Lastly, Western blot analysis of EV samples obtained from equal number of live cells was performed for EV enriched proteins. **b** Relative amount of EV protein normalised to 10^5^ MDA-MB-231 cells is shown. **c** Relative number of particles normalised to 2.5 × 10^6^ MDA-MB-231 cells is depicted. **d** Western blot analysis of EV enriched proteins Alix and TSG101 in EV samples obtained from 15 × 10^6^ MDA-MB-231 cells. Representative image shown, *n* = *3* biologically independent experiments. **e** Relative amount of EV protein normalised to 10^5^ 4T1 cells is shown. **f** Relative number of particles normalised to 2.5 × 10^6^ 4T1 cells is depicted. **g** Western blot analysis of EV enriched proteins Alix in EV samples obtained from 15 × 10^6^ 4T1 cells. Representative image shown, *n* = *3* biologically independent experiments. **h** Relative number of particles normalised to 2.5 × 10^6^ 4T1 cells is depicted. All data are represented as mean ± s.e.m. *n* = 3 biologically independent experiments, statistical significance was determined by paired two-tailed *t*-test. EVs were isolated by protocol 1 (**b**–**g**) and 2 (**h**). Full uncropped images for western blotting is provided in Supplementary Fig. 1.

Even though we followed the exact protocols as reported by the authors, we acknowledge that there are several variables that are different between the studies. For instance, the cell lines used in the two studies are presumably from different passages and could have genetically diverged upon continuous culture. Similarly, the FBS, conditioned media, NTA device and software used in the two studies are different. Another discrepancy between the two studies is that there was a 10-fold difference in EV protein amounts secreted by MDA-MB231 cells (7 µg per million cells was reported by Baek group while 0.7 µg per million cells was reported by Mathivanan group). However, for drug incubation studies, a general trend of either inhibition or augmentation of EV release should have been observed regardless of these variables. For instance, the ceramide inhibitor GW4869 has been reported to decrease the release of EVs by several groups regardless of isolation protocols and cell culture variables.

Overall, we report that SFX does not reduce the release of EVs (three independent researchers) and emphasise caution in using SFX (Merk 31739) as a drug to block EV release. However, we do acknowledge the fact that our findings do not challenge the authors main conclusion of SFX mediated reduction of primary tumour burden and metastasis though our results suggest that the phenotype observed may not be cancer cell-derived EV mediated. Further research is needed to understand as how SFX can reduce primary tumor burden and inhibit metastasis.

## Methods

### Cell culture

Human breast cancer cell line MDA-MB-231 was obtained from ATCC and murine breast cancer cell line 4T1 was gifted by Dr. Belinda Parker (La Trobe University). Cells were cultured at 37 °C, 5% CO_2_, and 95% humidity in the presence of 10% fetal calf serum (FCS - Assay Matrix #ASFBS) and 100 Unit/mL penicillin-streptomycin (GIBCO, Life Technologies). Alpha minimum essential medium (GIBCO, Life Technologies) was used for growing 4T1 cells whereas MDA-MB-231 cells were grown in Dulbecco’s modified Eagle’s medium (GIBCO, Life Technologies).

### Isolation of EVs

For protocol 1 (Fig. 1), EVs were isolated by exactly following the method reported by Im et al.^13^. Briefly, 4T1 and MDM-MB-231 cells were cultured until 80% confluence and were washed with 1× PBS and incubated in vehicle or SFX (Merck #31739) for 24 h in serum-free media. The conditioned media was then subjected to differential centrifugation (300 × *g* for 3 min, 2500 × *g* for 20 min and 10,000 × *g* for 30 min). Next, the supernatant was filtered using 0.2 μm filter followed by a centrifugation at 120,000 × *g* for 90 min. The EV pellet was then resuspended in PBS and subjected to another centrifugation at 120,000 × *g* for 90 min. The obtained EV pellet was then resuspended in PBS for further experiments.

For protocol 2 (Fig. 1), EVs were isolated with slight modifications from protocol 1 (differential centrifugation 2500 × *g* for 15 min and sEV incubated for 1 h at 4 °C). The modifications in protocol 2 as compared to protocol 1 is denoted with * in Fig. 1b.

For protocol 3, EVs were isolated by Mathivanan laboratory methods as described previously^15^. Briefly, conditioned media was collected and centrifuged at 500 × *g* for 10 min to remove cell debris followed by 2000 × *g* for 20 min at 4 °C. The supernatant was subjected to centrifugation at 10,000 × *g* for 30 min at 4 °C to remove large extracellular vesicles. The supernatant was then subjected to ultracentrifugation at 100,000 × *g* (SW28 rotor, Beckman) for 1 h at 4 °C. This step was repeated to wash the pellet with 1× PBS to collect sEVs for further analysis.

### Protein quantification

Equal volumes of EV samples were suspended in sodium dodecyl sulphate (SDS) buffer (8% w/v SDS, 10% v/v glycerol, and 0.4% v/v bromophenol blue, 200 mM Tris‐hydrochloride (Tris‐HCl), pH 6.8) with 100 mM dithiothreitol (DTT)). The protein samples were then heated at 92 °C for 2 min. The samples were resolved in SDS-PAGE at 150 V for 1 h followed by overnight SYPRO ruby staining according to manufactures instructions (Invitrogen™- S12000). Gels were then scanned using Typhoon™ FLA 9500 (GE Healthcare Life Sciences). Densitometric analysis was performed through ImageQuant™ to determine the protein concentration relative to a BenchMark™ Protein Ladder (Invitrogen). The obtained protein amount was then normalised to equal number of cells (1 × 10^5^).

### Nanoparticle tracking analysis (NTA)

EVs were analysed using NanoSight N300 (Malvern Instruments, Malvern, UK). The samples were monitored with the laser Monochromatic laser beam at 405 nm. EV sample volume was normalised to equal number of MDA-MB-231 and 4T1 (2.5 × 10^6^) cells. Three videos of 30 s each were taken for each sample (replicate). All the parameters and settings were maintained to be constant throughout the experiment (camera level at 12, detection threshold at 5 and syringe pump speed at 50 and temperature at 25 °C). Results obtained were analysed using NTA software 3.0 (ATA Scientific).

### Western blotting analysis

EV sample were normalised to equal number of cells (15 × 10^6^) before subjecting to lysis in SDS sample buffer. The protein samples were then resolved in SDS-PAGE at 150 V for 1 h. The resolved proteins were transferred onto a nitrocellulose membrane (Thermo Scientific™) using a wet transfer system at 25 V for 2.5 h in transfer buffer (11.5 mM Tris, 95 mM Glycine, 20% (v/v) Methanol). The membrane was blocked using 10% (w/v) skim milk in TTBS (100 mM Tris-HCl pH 7.5, 150 mM NaCl, 0.05% (v/v) Tween 20) for 1 h at room temperature. Next, the membrane was washed three times (10 min each) with TTBS before incubating in primary antibodies Alix (Cell Signaling Technology #2171S) and TSG101 (BD Transduction Laboratories #612696) over night at 4 °C (1:1000 dilution). Same wash cycle was applied following incubation with secondary antibody (IRDye 800CW secondary anti-mouse - LI-COR^®^) at room temperature for 1 h (1:10,000 dilution). Membrane was then washed three times with TTBS prior to visualisation using Odyssey® CLx (LI-COR^®^).

### Reporting summary

Further information on research design is available in the Nature Research Reporting Summary linked to this article.

## Supplementary information

Supplementary Information

Reporting Summary

## Data Availability

The data supporting the findings of the study are available in the Article or available from the authors upon request. Source data are provided with this paper.

## Source data

Source Data
