# Supplementary figures and images for: Sulfisoxazole does not inhibit the secretion of small extracellular vesicles
Source: Nat Commun. 2021 Feb 12;12:977. doi: 10.1038/s41467-021-21074-x (PMC7881022; doi:10.1038/s41467-021-21074-x)

## Full blots for Figure 2

**d**

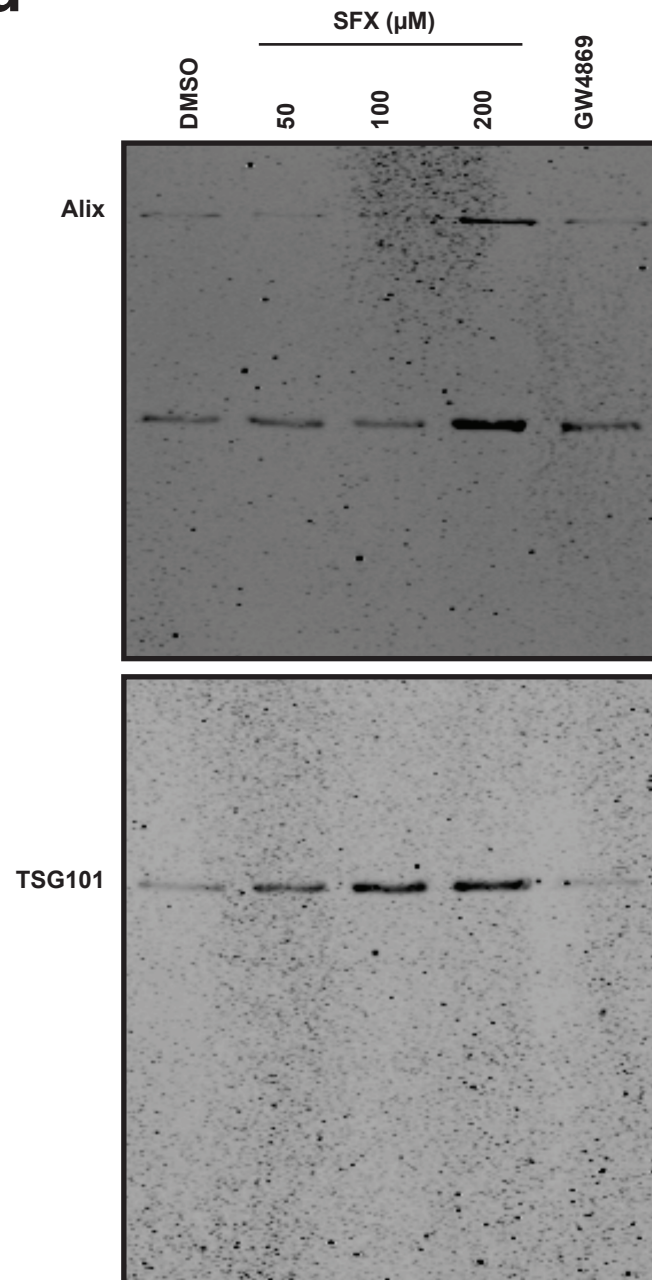

**e**

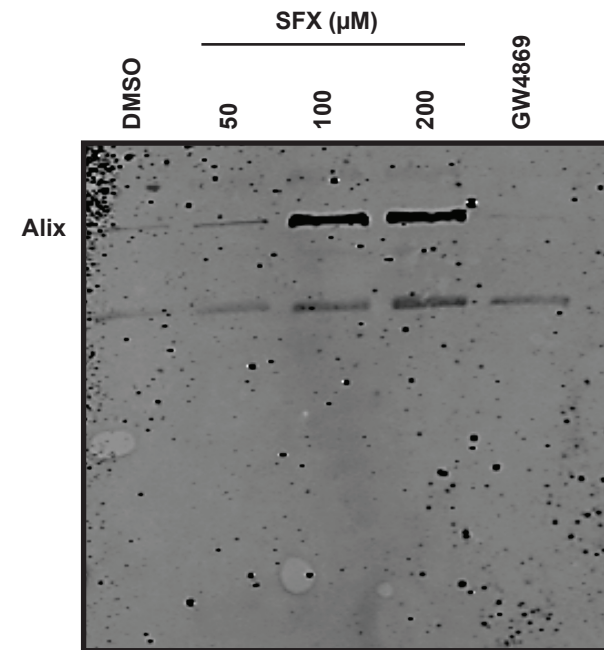

Supplement: Supplementary file 1 — Supplementary Information [file 41467_2021_21074_MOESM1_ESM.pdf]
