# Supplementary material for: Sulfisoxazole does not inhibit the secretion of small extracellular vesicles
Source: Nat Commun. 2021 Feb 12;12:977. doi: 10.1038/s41467-021-21074-x (PMC7881022; doi:10.1038/s41467-021-21074-x)
Supplement: Supplementary file 2 — Reporting Summary [file 41467_2021_21074_MOESM2_ESM.pdf]

nature research

Corresponding author(s): Suresh Mathivanan

Last updated by author(s): Sep 16, 2020

Reporting Summary

Nature Research wishes to improve the reproducibility of the work that we publish. This form provides structure for consistency and transparency in reporting. For further information on Nature Research policies, see our Editorial Policies and the Editorial Policy Checklist.

Statistics

For all statistical analyses, confirm that the following items are present in the figure legend, table legend, main text, or Methods section.

n/a	Confirmed	
☐	☒	The exact sample size (*n*) for each experimental group/condition, given as a discrete number and unit of measurement
☐	☒	A statement on whether measurements were taken from distinct samples or whether the same sample was measured repeatedly
☐	☒	The statistical test(s) used AND whether they are one- or two-sided<br>*Only common tests should be described solely by name; describe more complex techniques in the Methods section.*
☒	☐	A description of all covariates tested
☒	☐	A description of any assumptions or corrections, such as tests of normality and adjustment for multiple comparisons
☐	☒	A full description of the statistical parameters including central tendency (e.g. means) or other basic estimates (e.g. regression coefficient) AND variation (e.g. standard deviation) or associated estimates of uncertainty (e.g. confidence intervals)
☐	☒	For null hypothesis testing, the test statistic (e.g. *F*, *t*, *r*) with confidence intervals, effect sizes, degrees of freedom and *P* value noted<br>*Give P values as exact values whenever suitable.*
☒	☐	For Bayesian analysis, information on the choice of priors and Markov chain Monte Carlo settings
☒	☐	For hierarchical and complex designs, identification of the appropriate level for tests and full reporting of outcomes
☒	☐	Estimates of effect sizes (e.g. Cohen's *d*, Pearson's *r*), indicating how they were calculated

Our web collection on statistics for biologists contains articles on many of the points above.

Software and code

Policy information about availability of computer code

Data collection	Nanosight NTA Version 3.2
Data analysis	EV analysis - Nanosight NTA Version 3.2<br>Data - Microsoft Excel Version 2008<br>Statistics - GraphPad Prism Version 8.4.2<br>Western blot - Odyssey image studios Version 3.1<br>Protein gel - ImageQuant TL Version 8.1

For manuscripts utilizing custom algorithms or software that are central to the research but not yet described in published literature, software must be made available to editors and reviewers. We strongly encourage code deposition in a community repository (e.g. GitHub). See the Nature Research guidelines for submitting code & software for further information.

Data

Policy information about availability of data

All manuscripts must include a data availability statement. This statement should provide the following information, where applicable:

- Accession codes, unique identifiers, or web links for publicly available datasets
- A list of figures that have associated raw data
- A description of any restrictions on data availability

Further information and requests for data should be directed to and will be fulfilled by the Lead Contact, Suresh Mathivanan (S.Mathivanan@latrobe.edu.au).

Field-specific reporting

Please select the one below that is the best fit for your research. If you are not sure, read the appropriate sections before making your selection.

[x] Life sciences   [ ] Behavioural & social sciences   [ ] Ecological, evolutionary & environmental sciences

For a reference copy of the document with all sections, see nature.com/documents/nr-reporting-summary-flat.pdf

Life sciences study design

All studies must disclose on these points even when the disclosure is negative.

Sample size	No no sample size calculation was performed. The study was performed with 2 cell lines. Each experiment was conducted with 3 different technical replicates for one biological replicate. Experiments were performed in at least 3 biological replicates. It is regarded that 3 biological replicates are sufficient to report a biological phenotype in cell culture conditions.
Data exclusions	No data excluded.
Replication	The experiments were performed at least three times independently with three technical replicates each. All attempts at replication were successful.
Randomization	Samples were allocated to a group based on treatment with or without drug.
Blinding	No blinding was done, but three colleagues performed the experiments independently without being influenced by each other. We normally do not perform blinding experiments for cell culture conditions and the same individual performs the cell culture, treatment and processing.

Reporting for specific materials, systems and methods

We require information from authors about some types of materials, experimental systems and methods used in many studies. Here, indicate whether each material, system or method listed is relevant to your study. If you are not sure if a list item applies to your research, read the appropriate section before selecting a response.

Materials & experimental systems

n/a	Involved in the study	
[ ]	[x]	Antibodies
[ ]	[x]	Eukaryotic cell lines
[x]	[ ]	Palaeontology and archaeology
[x]	[ ]	Animals and other organisms
[x]	[ ]	Human research participants
[x]	[ ]	Clinical data
[x]	[ ]	Dual use research of concern

Methods

n/a	Involved in the study	
[x]	[ ]	ChIP-seq
[x]	[ ]	Flow cytometry
[x]	[ ]	MRI-based neuroimaging

Antibodies

Antibodies used	Alix (Cell Signaling Technology #2171S)<br>TSG101 (BD Transduction Laboratories #612696)<br>IRDye 800CW secondary anti-mouse (LI-COR #926-32210)
Validation	All antibodies have been used according to manufacturer's instructions. For details of verification, relevant citations or further information see the manufacturer's websites.

Eukaryotic cell lines

Policy information about cell lines

Cell line source(s)	Human breast cancer cell line MDA-MB-231 was obtained from ATCC<br>Murine breast cancer cell line 4T1 was obtained from ATCC and gifted by Dr. Belinda Parker (La Trobe University).
Authentication	The cell lines used were not authenticated. MDA-MB-231 and 4T1 cells were purchased from ATCC.
Mycoplasma contamination	The cell lines were negative for mycoplasma contamination.
Commonly misidentified lines (See ICLAC register)	No commonly misidentified cell lines were used in the study.
